# Novel 1.5 GPa-strength with 50%-ductility by transformation-induced plasticity of non-recrystallized austenite in duplex steels

**DOI:** 10.1038/s41598-017-01514-9

**Published:** 2017-04-28

**Authors:** Seok Su Sohn, Hyejin Song, Min Chul Jo, Taejin Song, Hyoung Seop Kim, Sunghak Lee

**Affiliations:** 1 Center for Advanced Aerospace Materials, Pohang University of Science and Technology, Pohang, 790-784 Korea; 2 HIMASS research project team, Technical Research Laboratories, POSCO, Kwangyang, 545-875 Korea

## Abstract

Needs for steel designs of ultra-high strength and excellent ductility have been an important issue in worldwide automotive industries to achieve energy conservation, improvement of safety, and crashworthiness qualities. Because of various drawbacks in existing 1.5-GPa-grade steels, new development of formable cold-rolled ultra-high-strength steels is essentially needed. Here we show a plausible method to achieve ultra-high strengths of 1.0~1.5 GPa together with excellent ductility above 50% by actively utilizing non-recrystallization region and TRansformation-Induced Plasticity (TRIP) mechanism in a cold-rolled and annealed Fe-Mn-Al-C-based steel. We adopt a duplex microstructure composed of austenite and ultra-fine ferrite in order to overcome low-yield-strength characteristics of austenite. Persistent elongation up to 50% as well as ultra-high yield strength over 1.4 GPa are attributed to well-balanced mechanical stability of non-crystallized austenite with critical strain for TRIP. Our results demonstrate how the non-recrystallized austenite can be a metamorphosis in 1.5-GPa-grade steel sheet design.

## Introduction

Reduction in vehicle weight becomes more emphasized for reducing exhaust emission and improving fuel efficiency^1–3^. In addition to lightweight needs, automotive steels require an excellent combination of strength and ductility as well as improvement of safety and crashworthiness qualities. Particularly in applications for structural reinforcement components composed of pillars, side sills, and front cross members, ultra-high yield strength over 1 GPa and sufficient stiffness are required. These Giga-grade automotive sheet markets are largely divided by cold-rolled sheets and hot press forming (HPF) sheets, and HPF sheets are used alone in 1.5-GPa-grade applications. HPF sheets are hardened by boron addition and martensitic transformation during the HPF process, and their resultant tensile strength reaches 1.5 GPa at elongation of about 6%^4^. However, their applications have been limited by energy consumption due to heating procedures, reduction in productivity due to prolonged processing time, and micro-cracking due to corrosion protection coating^4, 5^. If well-formable cold-rolled 1.5-GPa-grade sheets are successfully developed, thus, they have merits of excellent formability, weldability, and surface quality enough to complement drawbacks of HPF steels.

Austenitic or (ferrite + austenite) duplex microstructures are nominated as cold-rolled 1.5-GPa-grade sheets because of potentials of various strengthening mechanisms such as TRansformation-, TWinning-, and Micro Band-induced plasticity (TRIP, TWIP, and MBIP, respectively)^6–13^. High-Mn TWIP steels show excellent tensile strength and ductility over 1 GPa and 60%, respectively, but their yield strength ranges from 0.2 to 0.6 GPa because of austenite matrix characteristics^14–18^. Thus, the full utilization of austenite might not be a proper approach. In duplex steels, the austenite is transformed to α′-martensite during deformation, which leads to excellent tensile strength (1200 MPa) and elongation (30%), depending on composition, size, orientation, distribution, and stacking fault energy of austenite. In order to further enhance the strength and ductility, many researchers have been focused on grain refinement^19, 20^, alloy carbide precipitation^14, 21–23^, and κ-carbide precipitation^12, 24, 25^, but it is not easy to obtain the yield strength over 1 GPa and enough ductility.

As a promising method to achieve ultra-high strengths of 1.0~1.5 GPa together with excellent ductility above 50%, utilization of non-recrystallization region in a cold-rolled and annealed Fe-Mn-Al-C-based steel (composition; Fe-0.5C-11.8Mn-2.9Al-1Si-0.32Mo-0.5 V (wt.%)) has been suggested in the present study. Cold-rolled sheets are generally recrystallized by the annealing because the existence of non-recrystallized region seriously deteriorates the ductility^26–28^. Here we show a plausible method of improving ductility as well as ultra-high strength by actively utilizing the non-recrystallization region in combination with TRIP mechanism. We adopt a duplex microstructure composed of austenite and ultra-fine ferrite in order to overcome low-yield-strength characteristics of austenite. When mechanical stability of non-crystallized austenite is well balanced with critical strain for TRIP, stress and strain balance can be sustained in a very wide range of plastic deformation without tensile necking. Complex microstructures, which are mixed with recrystallized and non-crystallized regions and transformed martensite, greatly improve both strength and ductility. Our results demonstrate how the non-recrystallized austenite can be a metamorphosis in 1.5-GPa-grade steel sheet design.

## Results and Discussion

### Duplex microstructures

**Figure 1:**
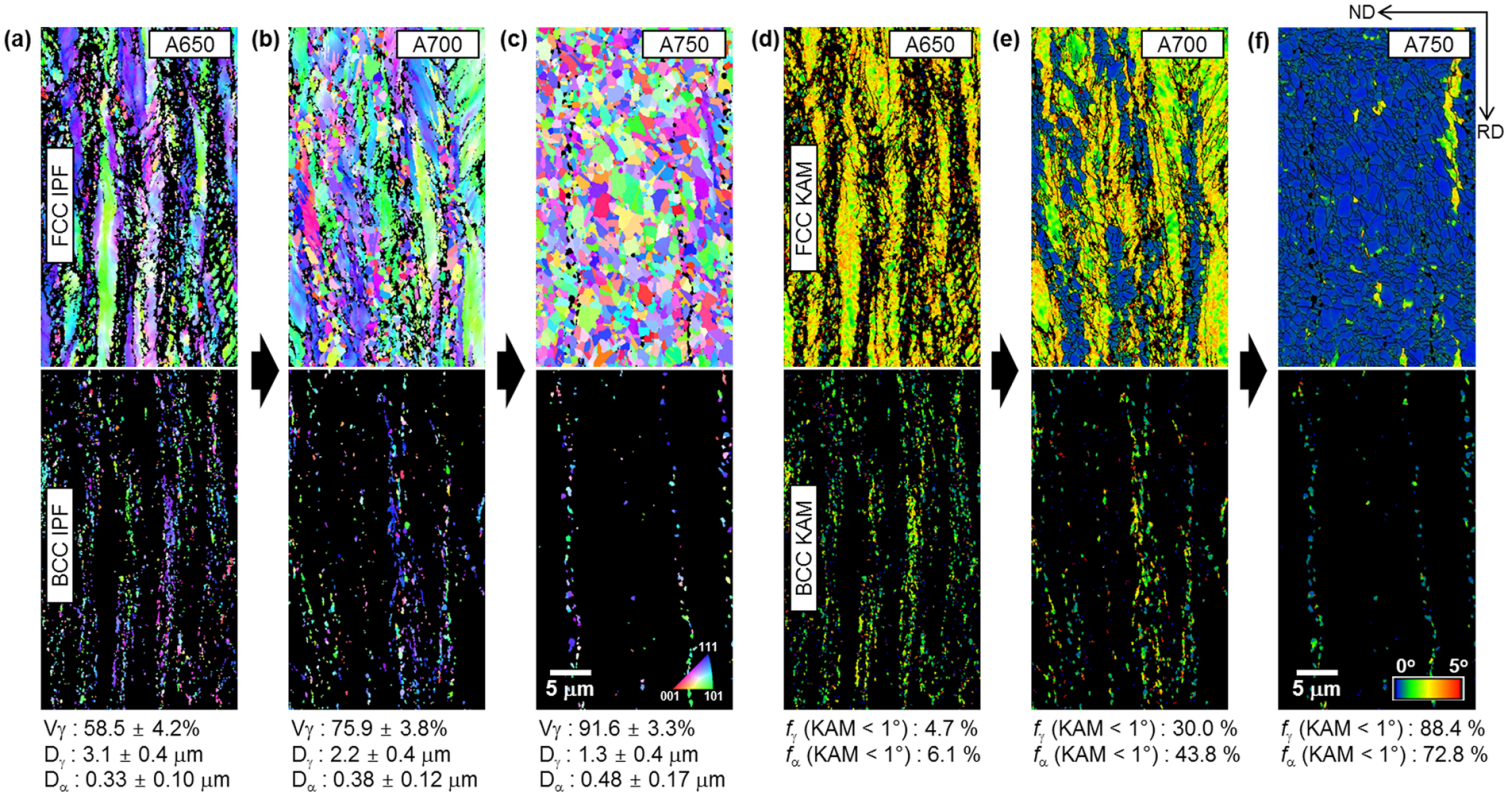
Microstructures of annealed duplex steels. (**a**–**c**) EBSD inverse pole figure (IPF) maps of austenite and ferrite, together with grain sizes of austenite and ferrite (D_γ_ and D_α_). Austenite and ferrite grains are elongated parallel to the rolling direction, and ferrite grains are extremely small. (**d**–**f**) EBSD kernel average misorientation (KAM) maps of austenite and ferrite. High KAM valued regions containing elongated grains and low KAM valued regions containing equi-axed grains indicate non-recrystallized and recrystallized regions, respectively.

The steel sheets annealed at 650, 700, and 750 °C are referred to as “A650”, “A700”, and “A750”, respectively, for convenience. EBSD inverse pole figure (IPF) maps of austenite and ferrite, together with grain sizes of austenite and ferrite (D_γ_ and D_α_) and volume fractions of austenite (V_γ_) measured with XRD analysis^29^, are shown in Fig. 1(a–c). Austenite grains in the A650 steel are elongated parallel to the rolling direction, and their average size is 3.1 μm. Ferrite grains are extremely small (0.33 μm) in the A650 steel (Fig. 1(a)). Austenite grains are refined to 1.3~2.2 μm in the A700 and A750 steels, and have an equi-axed shape in the A750 steel. Ferrite grains are coarsened to 0.48 μm with increasing annealing temperature, while their volume fractions decrease (Fig. 1(b,c)).

EBSD kernel average misorientation (KAM) maps of austenite and ferrite are shown in Fig. 1(d–f). KAM was calculated up to the third neighbor shell with a maximum misorientation angle of 5°, and KAM maps were served as a measure of deformation-induced local orientation gradients^30^. Most of austenite grains in the A650 steel show high KAM values (Fig. 1(d)). As the annealing temperature increases, blue regions showing low KAM values (below 1°) increase (Fig. 1(e,f)). High KAM valued regions containing elongated grains and low KAM valued regions containing equi-axed grains indicate non-recrystallized and recrystallized regions, respectively. Partial recrystallization occurs in the A700 steel, while most of austenite grains are recrystallized in the A750 steel. KAM values of ferrite grains are also high in the A650 steel, and tend to decrease with increasing annealing temperature. Fractions of KAM based on 1°, which is regarded as sufficient occurrence of recrystallization^31^, were measured. The fractions of KAM of 1° or smaller for austenite are 4.7, 30.0, and 88.4% in the A650, A700, and A750 steels, respectively. This implies that most of grains are not recrystallized in the A650 steel, whereas they are recrystallized in the A750 steel.

**Figure 2:**
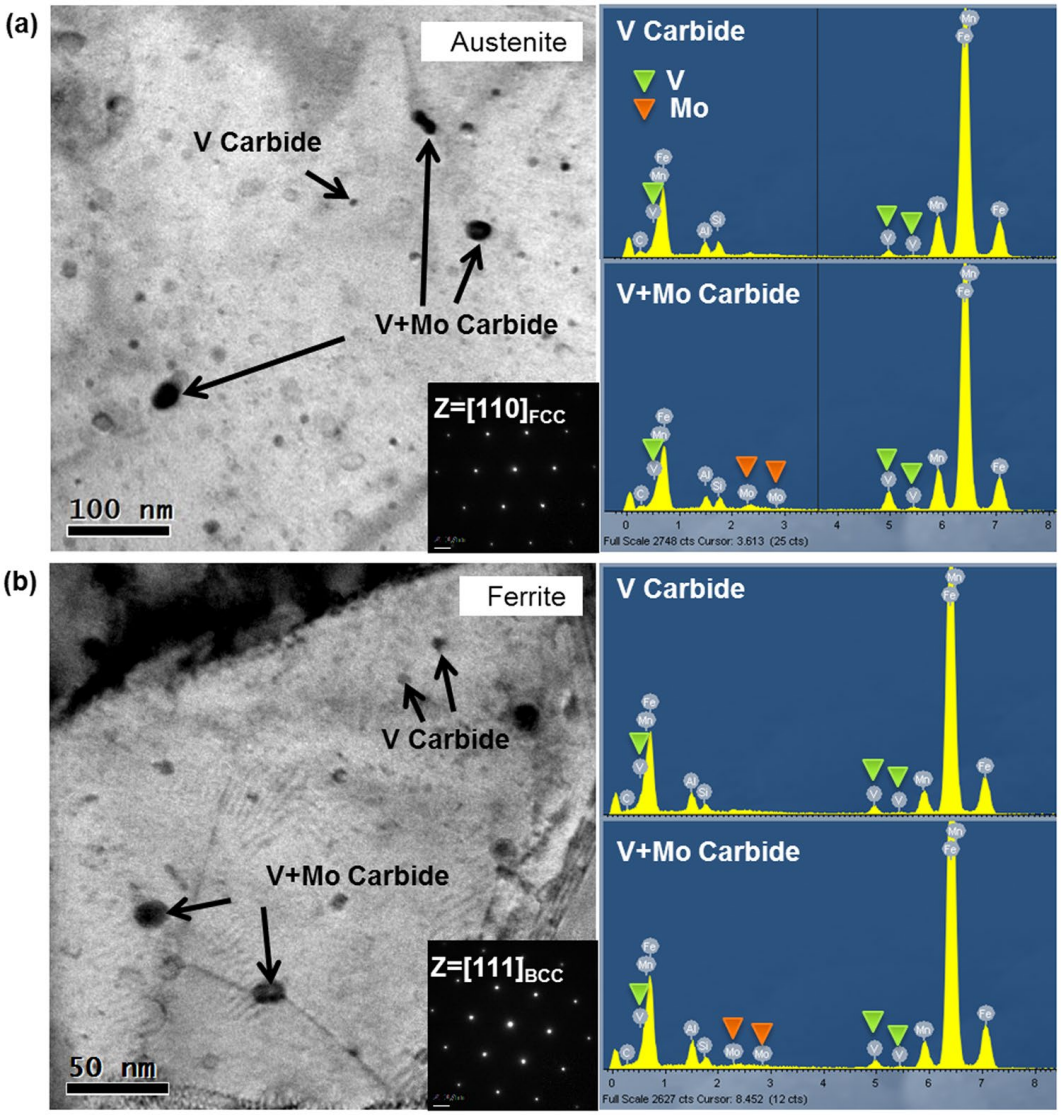
Carbide precipitation. (**a**) TEM bright-field (BF) image and energy-dispersive spectrometry (EDS) data for austenite in the A700 steel. (**b)** TEM BF image and EDS data for ferrite. Extremely fine precipitates (size; 5~10 nm) have the higher V content, which indicates that they are V carbides. Coarser precipitates (size; ~20 nm) having higher Mo and V contents are identified to be complex (V + Mo) carbides.

TEM bright-field images and energy-dispersive spectrometry (EDS) data for austenite and ferrite in the A700 steel are shown in Fig. 2(a,b). Extremely fine precipitates (size; 5~10 nm) have the higher V content, which indicates that they are V carbides. Coarser precipitates (size; ~20 nm) having higher Mo and V contents are identified to be complex (V + Mo) carbides. Relatively low density of carbides is shown in the ferrite.

### Novel tensile properties

**Figure 3:**
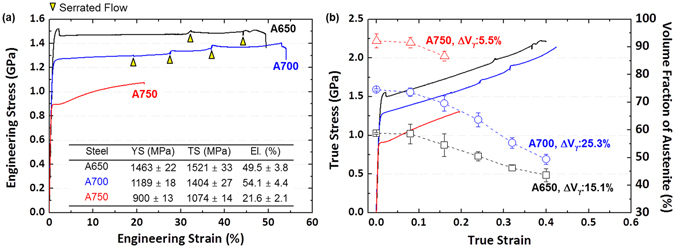
Room-temperature tensile properties and austenite volume fractions. (**a**) Room-temperature tensile engineering stress-strain curves and tensile properties. The A650 steel shows extremely high yield and tensile strengths (1.46 and 1.52 GPa, respectively) and high elongation of 50%. (**b**) True stress-strain curves (solid lines) and austenite volume fractions (dashed lines). The true tensile strengths of the A650 and A700 steels continuously increase to 2.1 GPa with increasing true strain.

Figure 3(a) shows room-temperature engineering stress-strain curves and tensile properties of the annealed steels. The A650 steel shows extremely high yield and tensile strengths (1.46 and 1.52 GPa, respectively) and high elongation of about 50%, and its tensile behavior shows a steady stress flow after the yield point. The yield and tensile strengths of the A700 steel are also very high (1.19 and 1.4 GPa, respectively), while the elongation reaches 54%. Its work hardening is larger than that of the A650 steel. A few serrated flows are observed as marked by yellow arrows. The A750 steel has the lowest yield and tensile strengths and elongation without serrated flows.

Figure 3(b) shows true stress-strain curves (solid lines) and volume fractions of austenite (dashed lines). The true tensile strengths of the A650 and A700 steels continuously increase to 2.1 GPa with increasing true strain, although engineering tensile curves show a steady stress flow (Fig. 3(a)). The volume fraction of austenite gradually decreases from 59 to 44% and from 75 to 49% in the A650 and A700 steels, respectively, which indicates the martensitic transformation occurring during the tensile deformation.

### Deformation mechanisms

**Figure 4:**
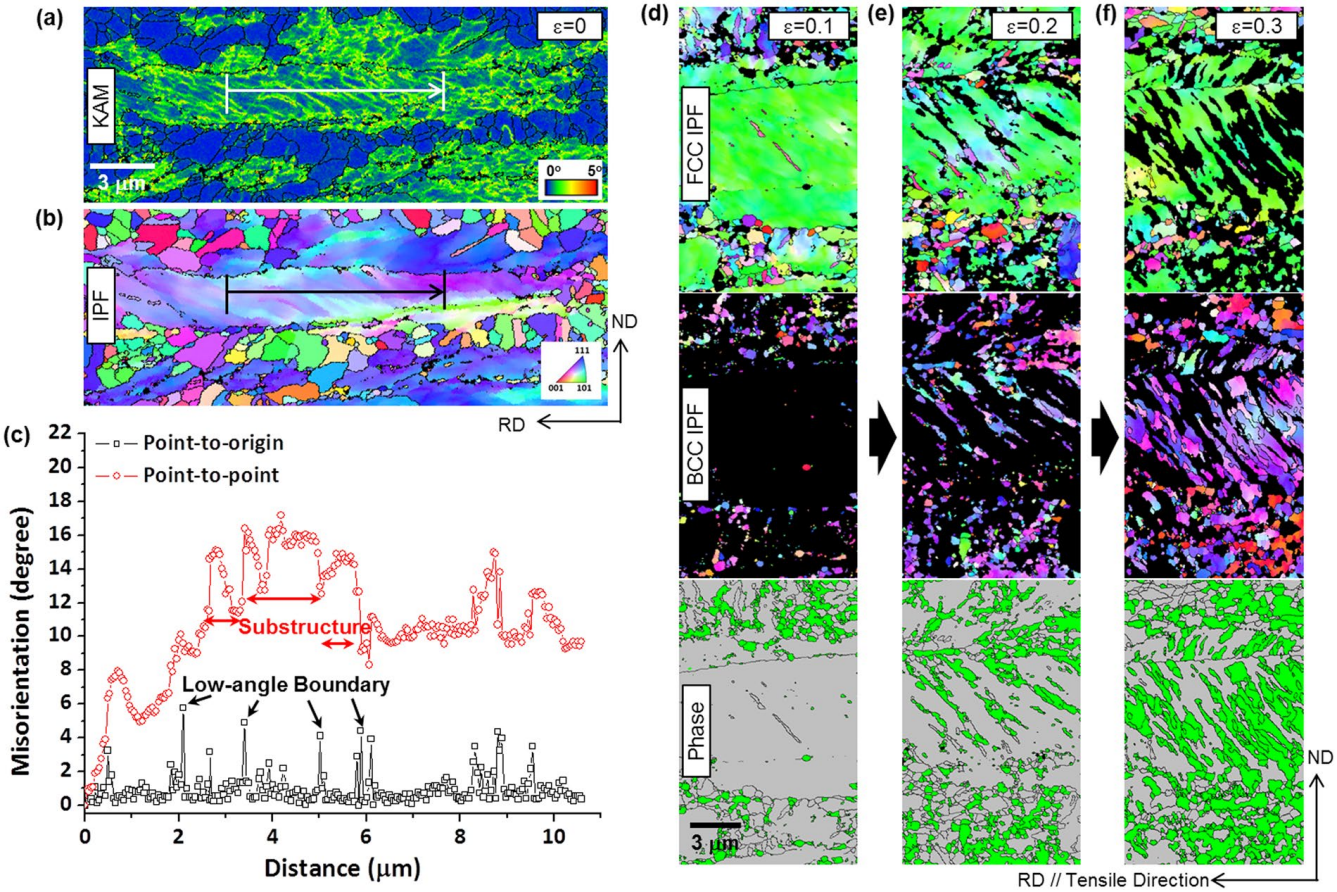
Substructure formation and TRIP behavior in non-recrystallized austenite. (**a–c**) EBSD KAM and IPF maps and misorientation profile data of the A700 steel before the tensile deformation. Substructures having low-angle boundaries are well developed. (**d–f**) IPF and phase maps of the areas deformed after the interrupted tensile test at true strains of 0.1, 0.2, and 0.3. Martensite is formed and grown at grain boundaries or along shear bands in non-recrystallized austenite grains.

In order to elucidate extremely high strengths over 1.4 GPa with elongation of 50%, detailed EBSD analyses were conducted. Figure 4(a,b) shows KAM and IPF maps and misorientation profile data of the A700 steel before the tensile deformation. In austenite grains of the non-recrystallized region, a few shear bands which are formed during the cold rolling are remained along the 45° direction deviated from the rolling direction (Fig. 4(a,b)). Accordingly, substructures having low-angle boundaries are well developed, as shown in the KAM map (Fig. 4(a)) and misorientation profile (Fig. 4(c)). The areas deformed after the interrupted tensile test at true strains of 0.1, 0.2, and 0.3 are shown in Fig. 4(d–f). At the true strain of 0.1, the austenite to α′-martensite transformation does not occur (Fig. 4(d)), which corresponds with the variation in austenite volume fraction in Fig. 3(b). At the strain of 0.2, the martensite starts to form at grain boundaries or along shear bands in non-recrystallized austenite grains, whereas they are hardly formed in recrystallized austenite grains (Fig. 4(e)). At the strain of 0.3, the martensite is grown in non-recrystallized austenite grains, while it is finely formed in recrystallized austenite grains (Fig. 4(f)). These results indicate that shear bands and substructures act as major nucleation sites of martensite, and that the TRIP occurs more readily in the non-recrystallized region than in the recrystallized region because the mechanical stability against TRIP is lower in non-recrystallized austenite grains^32^.

**Figure 5:**
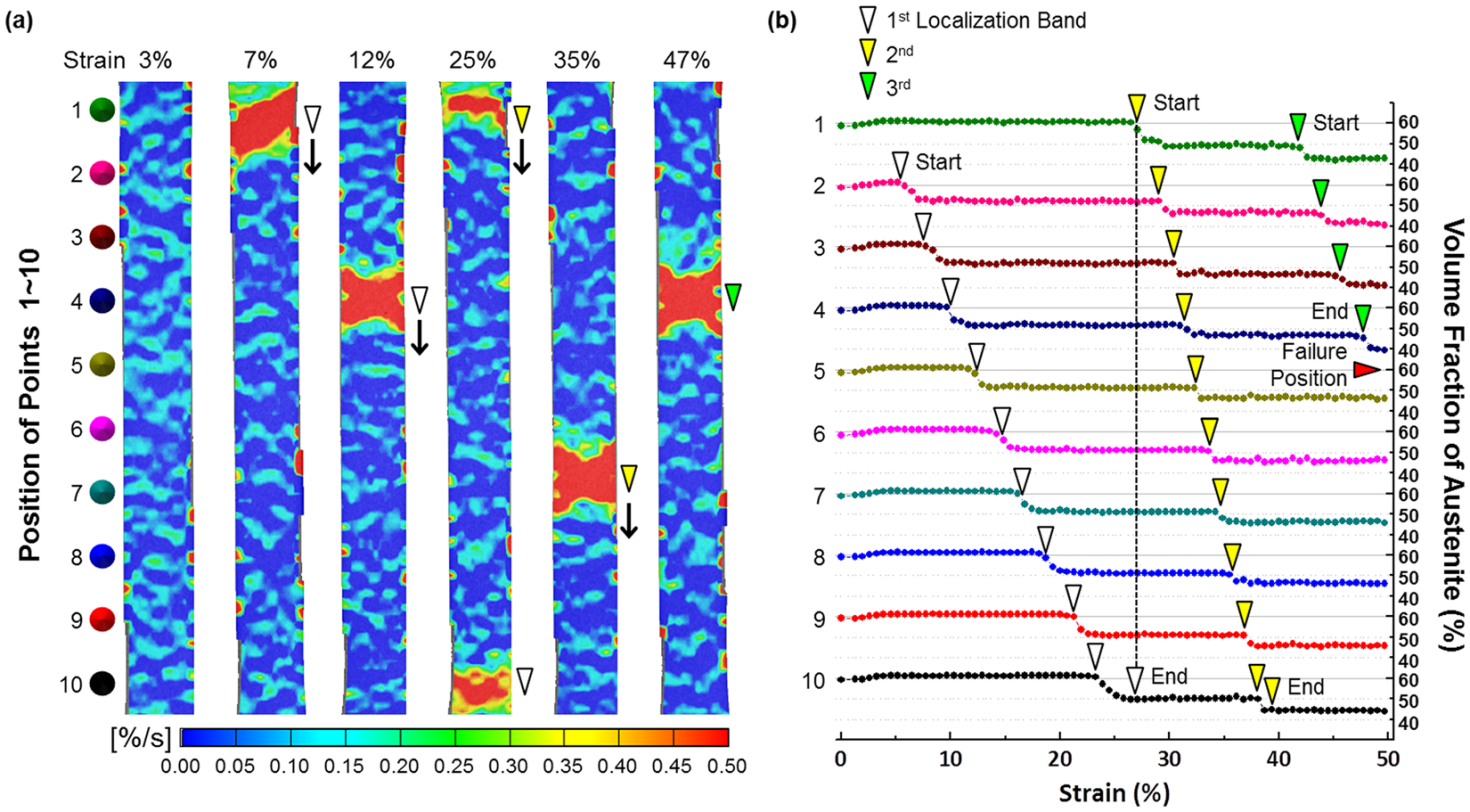
Strain rate distribution and localized martensitic transformation during deformation of the A650 steel. (**a**) Digital images of strain rate in strain range of 3~47% after points ‘1’~‘10’ were marked in the specimen gage section of the A650 steel. Three localized bands are generated, moved downward, and diminished as marked by white, yellow, and green arrows. (**b**) Variations in austenite volume fraction during the generation and movement of localized bands. The martensitic transformation occurs step by step while three localized bands are generated and diminished.

Figure 5(a,b) shows digital images of strain rate and volume fraction of austenite in strain range of 3~47% after points ‘1’~‘10’ were marked in the specimen gage section of the A650 steel. The austenite volume fraction was measured at every 0.03 mm in the gage section by a feritscope. At the strain of 3%, the A650 steel is homogeneously deformed. At the strain of 7%, strain localization occurs in a 45-deg-crossed mode between points 1 and 2, as marked by a white arrow in Fig. 5(a). At this time, the austenite volume fraction is rapidly reduced by about 10% (Fig. 5(b)). This 1^st^ localized band moves downward as the strain increases. While this band moves, the martensitic transformation occurs as marked by white arrows in Fig. 4(b), and the austenite volume fraction remains after the band passes through. At the strain of 25%, a new 2^nd^ band starts to move downward again at point 1 (yellow arrow) right after the 1^st^ band disappears. At point 1, 12% of martensite is formed while the 2^nd^ band passes, and 6% of martensite is formed at rests of points (points 2~10). The time of 2^nd^ band disappearance and 3^rd^ band start as well as the time of 1^st^ band disappearance and 2^nd^ band start are matched with serrated flow points (yellow arrow marks in Fig. 3(a)). During the deformation of 50% strain, three localized bands are formed and diminished, and the final failure occurs at points 4~5 as indicated by a red arrow. These digital image correlation (DIC) analyses plausibly explain the steady stress flow after the yield point and serrated flows in Fig. 3(a).

### Yield strength improvement mechanisms

The present duplex steels show novel tensile properties of extremely high tensile strength over 1.5 GPa with elongation of 50% in the A650 steel and 1.4 GPa with 54% in the A700 steel. It is also noted that their yield strengths are very high (1.19~1.46 GPa). Since very fine carbides such as V and V + Mo carbides are distributed inside austenite and ferrite grains (Fig. 2(a,b)), these steels basically show a precipitation strengthening. A substitutional solid-solution strengthening by adding 1 wt.% of Si is additionally expected^33^.

The more important factor than micro-alloying effects is a role of constituent phases of ferrite and austenite. The A650 steel consists of 40 vol.% of fine ferrite, whose size is ten-time smaller than the austenite grain size (~3 μm). Inside austenite grains, most of cold-rolled deformation is retained in a non-recrystallized state, and ferrite grains show a high lattice distortion (Fig. 1(a,d)). This indicates that extremely fine ferrite and non-recrystallized austenite favorably affect the improvement in yield strength up to 1.46 GPa. As the annealing temperature increases to 700~750 °C, the fraction of non-recrystallized region decreases to 70~12%, and the ferrite grain size increases to 0.4~0.5 μm. Accordingly, the yield strengths of the A700 and A750 steels decrease to 1.19 and 0.9 GPa, respectively.

### Utilization of TRIP in non-recrystallized austenite

When the cold-rolled deformation is retained, the tensile elongation would be very low, while the yield strength would be very high, because the further deformation cannot be readily accommodated^26–28^. In addition, the TRIP generally works as a major strengthening mechanism when the martensite is formed from the recrystallized austenite^34^. In the non-recrystallized region of the A650 steel where most of cold-rolled deformation is retained, thus, the powerful TRIP cannot be expected. However, the plastic deformation occurs continuously from the yield point to the strain of 50%. This is because the necking is suppressed by the TRIP along substructures formed in the non-recrystallized region (Fig. 4(a–c)). The steady stress flow behavior is a quite unique phenomenon (Fig. 3(a)), which implies that the persistent elongation is not simply explained by the TRIP.

According to the DIC data (Fig. 5(a,b)), a relatively small amount of martensite (less than 10%) is formed step by step, instead of homogeneous martensitic transformation, while localized bands pass through the specimen gage section. The stress-strain curve of the A650 steel (Fig. 3(a)) shows a serrated peak at the strain of 30% after the steady stress flow, which matches with the point of 1^st^ band disappearance and 2^nd^ band start. At this strain, the amount of martensitic transformation is twice larger than at the other strains because both 1^st^ and 2^nd^ bands exist simultaneously. At the strain of 2^nd^ band disappearance and 3^rd^ band start (45%), a serrated peak also appears. Since these serrated peak points are corresponded with points of instantaneous martensitic transformation, serrated flows are plausibly explained by the inhomogeneous step-by-step-type martensitic transformation.

In the present chemical composition of Fe-0.5C-11.8Mn-2.9Al-1Si-0.32Mo-0.5 V (wt.%), the critical strain required for TRIP is quite high. If the cold-rolled deformation retained in the non-recrystallized region is appropriately utilized, like in the A650 steel, the critical strain can be easily reached at a relatively low strain, and the martensite is well formed at austenite grains in the non-recrystallized region (Fig. 3). In the A700 steel whose volume fraction of non-crystallized region is 70%, the yield point disappears in the initial stage of deformation because the recrystallized region is homogeneously deformed. The strain energy is continuously accumulated even in the recrystallized region by the help of TRIP in the non-recrystallized region, which can exceed the deformation limit of recrystallized austenite. The sufficient strengthening effect of martensite formed in the recrystallized region on the austenite matrix as well as higher volume fractions of austenite and deformation-induced martensite provide the strengthening of +0.22 GPa after the yielding. This unique trend in the A650 and A700 steels, *i*.*e*., improvement of both strength and ductility, can be achieved by the appropriate stability of non-recrystallized austenite grains.

In the A750 steel mostly composed of the recrystallized region, on the other hand, the strain energy accumulated by the simple tensile deformation is not enough to reach the critical strain. The failure occurs before reaching the critical strain. It is interesting to note that its elongation is lowest, although it is expected to be highest because most of austenite grains are recrystallized (Fig. 1(c,f)). It is generally accepted that the elongation increases with increasing annealing temperature, whereas the strength decreases^26–28, 35^, but both the strength and elongation decrease in the A750 steel. This is because the TRIP is not sufficiently achieved (only about 6%, Fig. 3(b)) by the increase in austenite stability due to grain refinement^36–39^ as well as the disappearance of accumulated deformation due to sufficient recrystallization^31^.

### Comparison of present duplex steels with conventional ultra-high-strength steels

**Figure 6:**
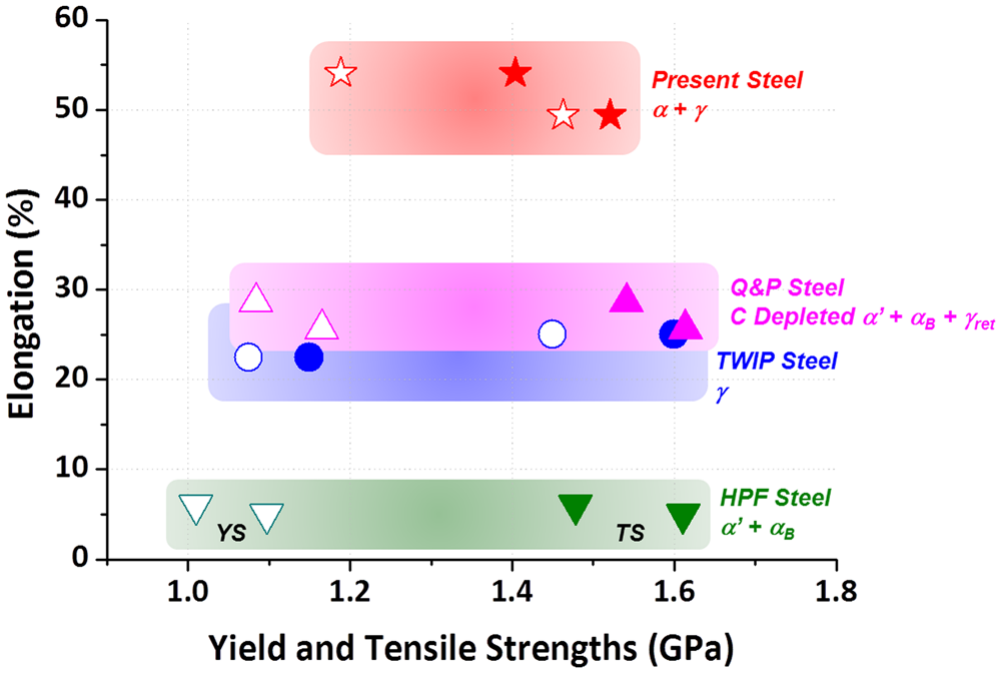
Comparison of present duplex steels with conventional ultra-high-strength steels. Room-temperature tensile properties and constituent phases of hot press forming (HPF) steels, TWinning-Induced Plasticity (TWIP) steels, and Quenching & Partitioning (Q&P) steels showing yield strength above 1 GPa. In HPF steels composed of full martensite matrix, the yield and tensile strengths are very high above 1.0 and 1.5 GPa, respectively, whereas their elongation is low below 8%. When nano-twinned structures are formed by relatively-low-temperature recovery treatments in high-Mn TWIP steels, the yield strength is enhanced above 1.0 GPa, but the elongation is reduced below 25%. Low~medium-Mn Q&P steels show ultra-high strengths by using the TRIP of retained austenite, while their elongation is lower than 30%.

The present duplex steels are compared with recently developed ultra-high-strength steels such as HPF, TWIP, quenching & partitioning (Q&P) steels showing yield strength above 1 GPa^4, 14–18, 28, 39–41^. Figure 6 shows room-temperature tensile properties of the ultra-high-strength steels, and their annealing conditions, constituent phases, and tensile properties are summarized in Table 1. In boron steels (*e*.*g*., HPF1; 22MnB5 and HPF2; 27MnCrB5) composed of full martensite matrix used for structural reinforcement components, the yield and tensile strengths are very high above 1.0 and 1.5 GPa, respectively, whereas their elongation is low below 8%^4^. High-Mn TWIP steels having excellent combination of strength and ductility show Giga-grade tensile strength, but their yield strength is quite low^14–18^. When nano-twinned structures are formed by relatively-low-temperature recovery treatments, the yield strength is enhanced above 1.0 GPa, but the elongation is reduced below 25%^28, 40^. Recently developed low~medium-Mn Q&P steels show ultra-high strengths by using the TRIP of retained austenite as well as containment of strong martensite and bainite, although they have disadvantages of low elongation below 30% and complex annealing conditions^41^. Attempts for achieving ultra-high strengths by utilizing hardened microstructures, precipitation strengthening, and recovery treatments generally encounter many difficulties to secure high elongation enough to form complex shapes. In view of excellent combination of strength and elongation, thus, the present duplex steels show good merits of high elongation above 50% as well as ultra-high yield and tensile strengths by effectively utilizing the TRIP of non-recrystallized austenite after the simple cold-rolling and annealing treatments.

**Table 1:** Annealing conditions, constituent phases, and room-temperature tensile properties of ultra-high-strength steels.

Steel Group	Annealing Condition	Constituent Phase	Yield Strength (MPa)	Tensile Strength (MPa)	Elongation (%)
Present 1	650 °C/10 min	γ + α	1436	1521	49.5
Present 2	700 °C/10 min	γ + α	987	1404	54.1
HPF 1^4^	950 °C/15 min	α′	987	1493	8.1
HPF 2^4^	900 °C/20 min	α′	1097	1611	4.0
TWIP 1^28^	550 °C/30 min	γ	1075	1150	22.5
TWIP 2^40^	500 °C/15 min	γ	1450	1600	25.0
Q&P 1^41^	880 °C/45 min + 360 °C/10~20 min	α′ + α_B_ + γ_ret_	1084	1541	28.6
Q&P 2^41^	Quenching + 360 °C/45 min	α′ + α_B_ + γ_ret_	1165	1614	25.6

## Conclusions

In conclusion, tensile properties of the present steels are quite outstanding, which have not been reported in previous studies on high-strength automotive steels. In particular, the present ultra-high yield strength is achieved mainly by the non-recrystallization of austenite, while it is also beneficially helped by the precipitation hardening of V and V + Mo carbide, solid solution hardening of Si, and ultra-fine ferrite grains with high dislocation density. The persistent elongation up to 50% is attributed to the TRIP in the non-recrystallization of austenite which can easily reach the critical strain for TRIP. This result is unexpected one because the deterioration of elongation in the non-crystallized region is generally accepted in commercial steels. However, the A650 steel shows a steady stress flow behavior because of inhomogeneous stage-by-stage-type deformation, which can achieve ultra-high yield and tensile strengths of 1.4~1.5 GPa together with excellent ductility of 50%. The alloying of 2.9 wt.% Al and 1 wt.% Si also leads to the weight reduction of about 6% in comparison to pure Fe^12^. The present duplex steels have superb properties of strength and ductility, easy manufacturing processes, and reduced weight, thus give a promise for new applications to multi-functional ultra-high-strength automotive steel sheets. However, the existence of non-crystallized region might negatively affect the formability or anisotropic mechanical properties (yield strength; 1557 MPa, tensile strength; 1605 MPa, elongation; 12.5% along the transverse direction in the A700 steel). In order to complement these drawbacks, intensive studies on new alloy and process designs for achieving the appropriate non-recrystallization of austenite on clarifying mechanisms involved in formability and anisotropic mechanical properties should be continued in the future.

## Methods

### Fabrication of materials

The steel used in this study was fabricated by a vacuum induction melting method, and its composition is Fe-0.5C-11.8Mn-2.9Al-1Si-0.32Mo-0.5 V (<0.02)(P + S) (wt.%). Si was added for solid solution strengthening, while V and Mo were added for precipitation strengthening^42–44^. After 45-mm-thick plates were homogenized at 1200 °C for 1 hour, they were hot-rolled between 1100 and 900 °C. They were then cooled in a furnace from 650 °C after holding at this temperature for 1 hour in order to simulate a coiling procedure. The hot-rolled 3-mm-thick sheets were rolled at room temperature to make 1-mm-thick sheets. The sheets were annealed at 650, 700, and 750 °C for 10 min, and were cooled in the air.

### Microstructure characterization

Phases present in the steel sheets were identified by X-ray diffraction (XRD, Cu K_α_ radiation, scan rate; 2 deg min^−1^, scan step size; 0.02 deg), transmission electron microscopy (TEM), and Electron back-scatter diffraction (EBSD) analysis. Their volume fractions were measured from the direct comparison method of XRD^29^ by using integrated intensities of (200)_α_ and (211)_α_ peaks and (220)_γ_ and (311)_γ_ peaks. For the TEM observation, sheets were mechanically polished to a thickness of 50 μm, punched to prepare disc specimens, and electro-polished in a solution of CH_3_COOH (90%) and HClO_4_ (10%) to prepare thin foils. The thin foils were observed by a transmission electron microscope (model; 2100, JEOL, Japan) operated at an acceleration voltage of 200 kV. The EBSD analysis (step size; 40 or 80 nm) was conducted by a field emission scanning electron microscope (FE-SEM, Quanta 3D FEG, FEI Company, USA). EBSD specimens were mechanically polished and electro-polished in a solution of CH_3_COOH (92%) and HClO_4_ (8%) at an operating voltage of 32 V for 15 sec. The data were then interpreted by orientation imaging microscopy (OIM) analysis software provided by TexSEM Laboratories, Inc.

### Mechanical property tests

Plate-type tensile specimens having gage length of 25 mm, gage width of 6 mm, and gage thickness of 1 mm were prepared in the longitudinal direction. They were tested at room temperature at a strain rate of 10^−3^ s^−1^ by a universal testing machine (model; 8801, Instron, Canton, MA, USA) of 100 kN capacity. The tensile test was conducted three times for each datum point. During the tensile test, volume fraction of austenite was measured by a magneto-inductive device, feritscope (model; MP30, Helmut Fischer GmbH & Co., Sindelfingen, Germany), and was corrected in consideration of measured values for geometrical or loading reasons (Villari-Effect)^45^.

### Digital imaging strain analysis

Digital image correlation (DIC) is a basic technique to measure the deformation amount including localized deformation strain during the tensile test^46–48^. Photographs of the tensile specimen surface were taken by two cameras (model: Phantom V7.3, Komi, Korea), and a vision strain gauge system (model; ARAMIS 5 M, GOM Optical Measuring Techniques, Germany) was used for detecting 3-dimensional coordinates of a deforming specimen surface. High-quality white- and black-color lacquers (model: Aqua, Motip Dupli, Germany) were sprayed on the longitudinal-transverse (L-T) plane of the tensile specimen to obtain random black-and-white speckled patterns, which were then used for the strain distribution analysis. This system recognized the surface structure in digital camera images, and allocated coordinates to image pixels.

## References

[1] Bouaziz O, Zurob H, Huang M (2013). Driving force and logic of development of advanced high strength steels for automotive applications. Steel Res. Int..

[2] Mayyas A, Qattawi A, Omar M, Shan D (2012). Design for sustainability in automotive industry: a comprehensive review. Renew Sustainable Energy Rev..

[3] Kuziak R, Kawalla R, Waengler S (2008). Advanced high strength steels for automotive industry. Arch. Civ. Mech. Eng..

[4] Karbasian H, Tekkaya AE (2010). A review on hot stamping. J. Mater. Process. Technol..

[5] Fan DW (2010). Coating degradation in hot press forming. ISIJ Int..

[6] De Cooman BC (2004). Structure-properties relationship in TRIP steels containing carbide-free bainite. Curr. Opin. Solid State Mater. Sci..

[7] Tirumalasetty GK (2012). Deformation-induced austenite grain rotation and transformation in TRIP-assisted steel. Acta Mater..

[8] Gibbs PJ (2011). Austenite stability effects on tensile behavior of manganese-enriched-austenite transformation-induced plasticity steel. Metall. Mater. Trans. A.

[9] Seo C-H (2012). Deformation behavior of ferrite-austenite duplex lightweight Fe-Mn-Al-C steel. Scr. Mater..

[10] Bouaziz O (2011). High manganese austenitic twinning induced plasticity steels: A review of the microstructure properties relationships. Curr. Opin. Solid State Mater. Sci..

[11] Gutierrez-Urrutia I, Raabe D (2011). Dislocation and twin substructure evolution during strain hardening of an e–22 wt.% Mn–0.6 wt.% C TWIP steel observed by electron channeling contrast imaging. Acta Mater..

[12] Frommeyer G, Brüx U (2006). Microstructures and mechanical properties of high-strength Fe-Mn-Al-C light-weight TRIPLEX steels. Steel Res. Int..

[13] Yoo JD, Hwang SW, Park K-T (2009). Origin of extended tensile ductility of a Fe-28Mn-10Al-1C steel. Metall. Mater. Trans. A.

[14] Scott C (2011). Precipitation strengthening in high manganese austenitic TWIP steels. Int. J. Mater. Res..

[15] Bayraktar E, Khalid FA, Levaillant C (2004). Deformation and fracture behavior of high manganese austenitic steel. J. Mater. Process. Technol..

[16] Bouaziz O (2011). Effect of chemical composition on work hardening of Fe-Mn-C TWIP steels. Mater. Sci. Technol..

[17] Lai HJ, Wan CM (1989). The study of work hardening in Fe-Mn-Al-C alloys. J. Mater. Sci..

[18] Liang X (2009). Microstructural evolution and strain hardening of Fe-24Mn and Fe-30Mn alloys during tensile deformation. Acta Mater..

[19] Sohn SS (2015). Novel ultra-high-strength (ferrite + austenite) duplex lightweight steels achieved by fine dislocation substructures (Taylor lattices), grain refinement, and partial recrystallization. Acta Mater..

[20] Ueji R (2008). Tensile properties and twinning behavior of high manganese austenitic steel with fine-grained structure. Scr. Mater..

[21] Mejíaa I (2014). Effect of Nb and Mo on the hot ductility behavior of a high-manganese austenitic Fe-21Mn-1.3Al-1.5Si-0.5C TWIP steel. Mater. Sci. Eng. A.

[22] Perrard F, Scott C (2007). Vanadium precipitation during intercritical annealing in cold rolled TIRP steels. ISIJ Int..

[23] Kang S, Jung J-G, Lee Y-K (2012). Effects of niobium on mechanical twinning and tensile properties of a high Mn twinning-induced plasticity steel. Mater. Trans..

[24] Choi K (2010). Effect of aging on the microstructure and deformation behavior of austenite base lightweight Fe-28Mn-9Al-0.8C steel. Scr. Mater..

[25] Gutierrez-Urrutia I, Raabe D (2013). Influence of Al content and precipitation state on the mechanical behavior of austenitic high-Mn low-density steels. Scr. Mater..

[26] Fonstein, N. *Advanced High Strength Sheet Steels*, 193–195 (Springer, 2015).

[27] Somani MC (2009). Enhanced mechanical properties through reversion in metastable austenitic stainless steels. Metall. Mater. Trans. A.

[28] Dini G, Najafizadeh A, Ueji R, Monir-Vaghefi SM (2010). Improved tensile properties of partially recrystallized submicron grained TWIP steel. Mater. Lett..

[29] Mahieu J, Maki J, De Cooman BC, Claessens S (2002). Phase transformation and mechanical properties of Si-free CMnAl transformation-induced plasticity-aided steel. Metall. Mater. Trans. A.

[30] Calcagnotto M, Ponge D, Demir E, Raabe D (2010). Orientation gradients and geometrically necessary dislocations in ultrafine grained dual-phase steels studied by 2D and 3D EBSD. Mater. Sci. Eng. A.

[31] Fang C, Garcia CI, Choi S, Deardo AJ (2015). A study of the batch annealing of cold-rolled HSLA steels containing niobium or titanium. Metall. Mater. Trans. A.

[32] Olson GB, Cohen M (1975). Kinetics of strain-induced martensitic nucleation. Metall. Trans. A.

[33] Serajzadeh S, Taheri AK (2002). An investigation on the effect of carbon and silicon on flow behavior of steel. Mater. Des..

[34] Jafarian H (2016). Characteristics of nano/ultrafine-grained austenitic TRIP steel fabricated by accumulative roll bonding and subsequent annealing. Mater. Charact..

[35] Kisko A (2016). Effect of reversion and recrystallization on microstructure and mechanical properties of Nb-alloyed low-Ni high-Mn austenitic stainless steels. Mater. Sci. Eng. A.

[36] Sohn SS (2013). Effect of annealing temperature on microstructural modification and tensile properties in 0.35C-3.5Mn-5.8Al lightweight steel. Acta Mater..

[37] van Dijk NH (2005). Thermal stability of retained austenite in TRIP steels studied by synchrotron X-ray diffraction during cooling. Acta Mater..

[38] Berrahmoune MR (2004). Analysis of the martensitic transformation at various scales in TRIP steel. Mater. Sci. Eng. A.

[39] Takaki S, Fukunaga K, Syarif J, Tsuchiyama T (2004). Effect of grain refinement on thermal stability of metastable austenitic steel. Mater. Trans..

[40] Zhou P, Liang ZY, Liu RD, Huang MX (2016). Evolution of dislocations and twins in a strong and ductile nanotwinned steel. Acta Mater..

[41] Gao G (2015). Enhanced strain hardening capacity in a lean alloy steel treated by a “distributed” bainitic austempering process. Acta Mater..

[42] Jacques PJ (2001). The developments of cold-rolled TRIP-assisted multiphase steels. Al-alloyed TRIP-assisted multiphase steels. ISIJ Int..

[43] Chen CY (2009). Precipitation hardening of high-strength low-alloy steels by nanometer-sized carbides. Mater. Sci. Eng. A.

[44] Michaud P (2007). The effect of the addition of alloying elements on carbide precipitation and mechanical properties in 5% chromium martensitic steels. Acta Mater..

[45] Behjati P, Kermanpur A, Najafizadeh A (2013). Application of martensitic transformation fundamentals to select appropriate alloys for grain refining through martensite thermomechanical treatment. Metall. Mater. Trans. A.

[46] Hong S (2014). Serration phenomena occuring during tensile tests of three high-manganese twinning-induced plasticity (TWIP) steels. Metall. Mater. Trans. A.

[47] Stinville JC (2015). High resolution mapping of strain localization near twin boundaries in a nickel-based superalloy. Acta Mater..

[48] Yan D, Tasan CC, Raabe D (2015). High resolution *in situ* mapping of microstrain and microstructure evolution reveals damage resistance criteria in dual phase steels. Acta Mater..

